# Quantification of the early pupillary dilation kinetic to assess rod and cone activity

**DOI:** 10.1038/s41598-021-88915-z

**Published:** 2021-05-05

**Authors:** Corinne Kostic, Sylvain V. Crippa, Lorette Leon, Christian Hamel, Isabelle Meunier, Aki Kawasaki

**Affiliations:** 1 Group for Retinal Disorder Research, Department of Ophthalmology, University of Lausanne, Hôpital Ophtalmique Jules Gonin, Lausanne, Switzerland; 2 Neuro-Ophtalmology, Department of Ophthalmology, University of Lausanne, Hôpital Ophtalmique Jules Gonin, Lausanne, Switzerland; 3 National Reference Centre for Inherited Sensory Diseases, Montpellier University Hospital,Institute for Neurosciences of Montpellier (INM), INSERM, University of Montpellier, Montpellier, France

**Keywords:** Physiology, Biomarkers, Neurology

## Abstract

Rods, cones and melanopsin contribute in various proportions, depending on the stimulus light, to the pupil light response. This study used a first derivative analysis to focus on the quantification of the dynamics of pupillary dilation that immediately follows light-induced pupilloconstriction in order to identify novel parameters that reflect rod and cone activity. In 18 healthy adults, the pupil response to a 1 s blue light stimulus ranging from − 6.0 to 2.65 log cd/m^2^ in dark-adapted conditions and to a 1 s blue light stimulus (2.65 log cd/m^2^) in light-adapted conditions was recorded on a customized pupillometer. Three derivative parameters which describe the 2.75 s following the light onset were quantified: dAMP (maximal amplitude of the positive peak), dLAT (latency of the positive peak), dAUC (area under the curve of the positive peak). We found that dAMP and dAUC but not dLAT have graded responses over a range of light intensities. The maximal positive value of dAMP, representing maximal rate of change of early pupillary dilation phase, occurs at − 1.0 log cd/m^2^ and this stimulus intensity appears useful for activating rods and cones. From − 0.5 log cd/m^2^ to brighter intensities dAMP and dAUC progressively decrease, reaching negligible values at 2.65 log cd/m^2^ indicative of a melanopsin-driven pupil response that masks the contribution from rods and cones to the early phase of pupillary dilation.

## Introduction

The retinal signal which initiates the pupil light reflex is primarily carried in the axons of a very small subset of retinal ganglion cells which express the photopigment melanopsin, the intrinsically photosensitive retinal ganglion cells (ipRGCs). The photoreceptive input to the retinal pupillomotor signal derives from rods, cones and the ipRGCs themselves as these are light sensitive cells capable of depolarisation via their own intrinsic, melanopsin-mediated phototransduction pathway. Light-activation of outer retinal rods and cones can extrinsically initiate depolarisation of ipRGCs via synaptic inputs, primarily through ON bipolar and amacrine cells^1^. These synaptic inputs from outer retinal photoreceptors expand the range of light intensities and temporal frequencies to which the ipRGCs will signal to central synaptic sites, including the pretectal olivary nucleus for mediating the pupil light reflex. Thus pupil light responses reflect the integration of light information detected by two fundamentally different photoreceptor systems, the outer retinal visual photoreceptors and the inner retinal melanopsin-based photoreception.

Understanding that the afferent pupillomotor signal is a hybrid of cone, rod and melanopsin responses to light has regenerated interest in colored light (chromatic) pupillometry because the possibility to evoke a pupil light reflex driven predominantly by a single photoreceptor holds appeal as a non-invasive tool to evaluate rod and cone function as well as melanopsin activity in children and in subjects unable to perform standard tests of vision^2,3^. For example, the pupil response to very dim (threshold) intensity light, regardless of wavelength, which is presented under dark-adapted conditions can be used as a biomarker of rod activation in isolation of cone and melanopsin contribution^4,5^. In a prior study, we had presented very dim short wavelength stimuli to healthy eyes and noted that subjects could perceived the light stimulus but did not see the blue color until brighter intensities were presented, hence when cones were recruited^6^. These rod-mediated pupil responses have been correlated with clinical disease in monogenetic retinal degeneration and hold promise as an alternative clinical tool for quantifying rod function.

Due to overlapping spectral sensitivities between rods, cones and melanopsin, use of monochromatic light stimuli, no matter how carefully selected, cannot isolate a single type of photoreceptor. However, combining spectral selection with other characteristics of the light stimulus that underscores the physiologic differences between outer vs inner retinal photoreception can favor activation of one vs the other, Charng et al. demonstrated that modulation of the stimulus duration could weighted the pupil response toward cone input^7^. Other investigators have demonstrated a correlation between the central perimetric function and the transient pupillary response to red light using latency to reach a criterion amplitude^8,9^. For isolation of the melanopsin–mediated pupil response in animal experiments, knockout models in small mammals have best addressed the confounding effect of integration between the photoreceptive systems^10,11^.

In a previous study, we used mouse knockout models to characterize features of the recorded pupil response to different colored lights which correlated with rod or cone or melanopsin function^12^. In addition to the more standard parameters of maximal contraction amplitude, sustained contraction amplitude and the post-illumination pupil response, we quantified the first derivative of the beginning of recording^13^. We were particularly interested in dissecting the initial fast response of the pupil to a light stimulus as a means to identify a response parameter that might define cone and rod contribution. We selected three parameters to define the initial pupil response. Two were well-known previously described parameters: the maximal constriction amplitude in the first 2 s following stimulus onset and the time to maximal constriction amplitude. We also measured a novel parameter which was a first derivative curve analysis of the first 2.5 s from light stimulus onset. The derivative curve describes the rate of change of constriction and dilation during the initial 3 s of the pupil response to a brief light stimulus. Pupillary constriction, a change toward a smaller pupil size, is indicated by negative values and the rate of change is indicated by the magnitude of these negative values. After reaching maximum rate of change of constriction, the derivative value briefly becomes zero as the pupil reaches its maximum constriction. Thereafter, if, the derivative values become positive, this corresponds to a dilation movement. A maximal dilation in the first 3 s of a light stimulus was found to be a purely rod and cone-driven phase of the pupil response in mice.

In this study, we apply the derivative analysis to the pupil recordings obtained in human subjects with normal eyes in order to understand the range and magnitude of the early derivative positive peak as a marker of rod and cone function.

## Results

### Pupil responses to blue light stimuli ranging from − 5 to 2.6 log cd/m^2^

**Figure 1:**
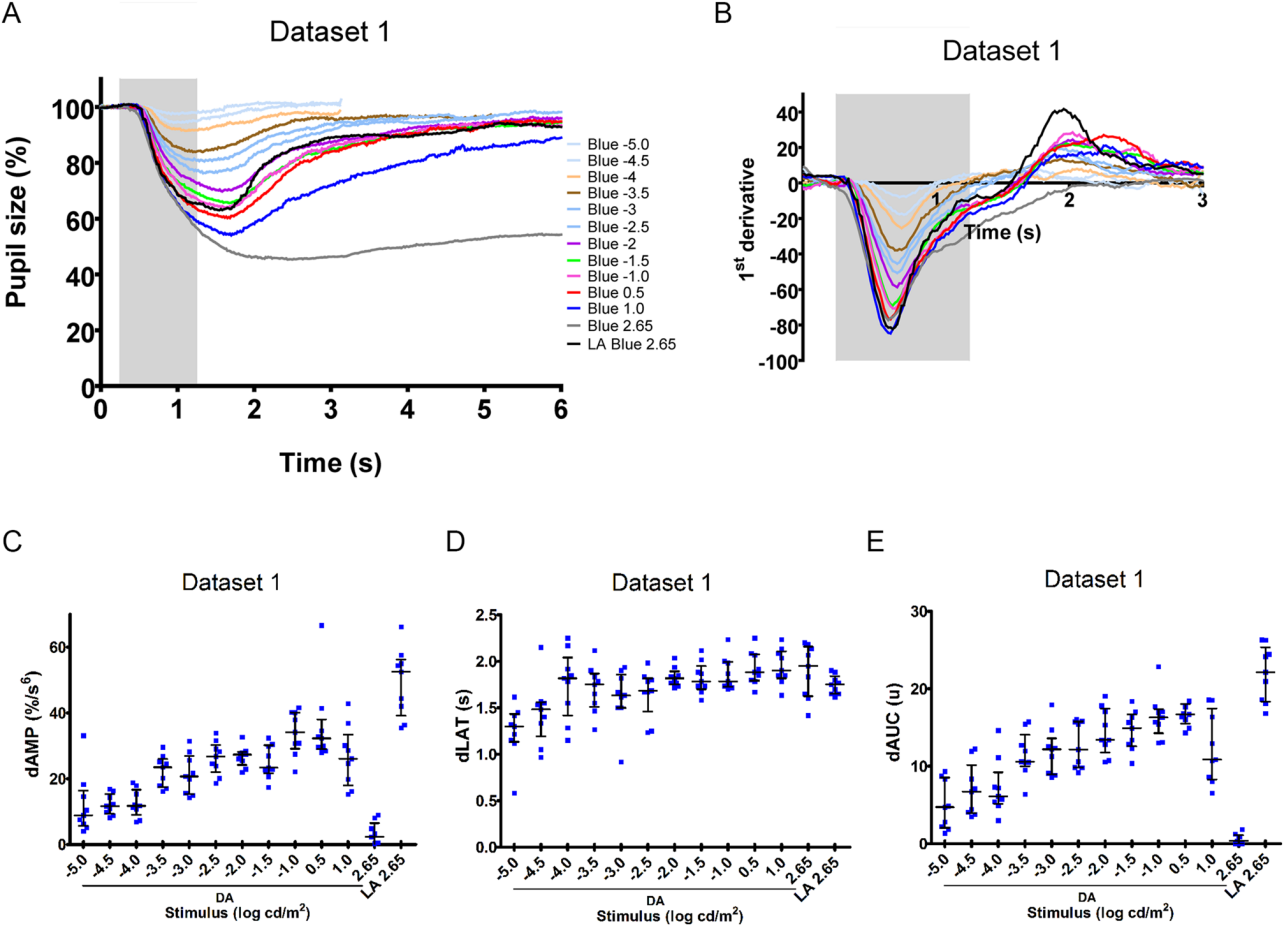
Pupil response curve and derivative analysis of Dataset 1 to blue light stimuli s presented under dark-adapted condition. (**A**) The mean relative pupil size (plain line) before, during and after a 1 s light stimulus is plotted against time (response curve). The different colors represent the different stimulus intensities from − 5 log cd/m^2^ to 2.65 log cd/m^2^. (**B**) The mean derivative responses plotted for the first 3 s of the pupil recordings shown in (**A**). LA Blue represents the pupil response to the blue light stimulus 2.65 log cd/m^2^ presented at the end of a light-adapted sequence. The shaded box represents the duration of the light stimulus. Three parameters were obtained by quantification of the derivative curve: the maximal positive peak amplitude (dAMP) (**C**), the latency of dAMP (**D**) and the area under the curve of the positive peak of the derivative (dAUC) (**E**) are presented as distribution plots. The median (long horizontal bar) and the interquartile range (short horizontal bar) are superposed at each stimulus intensity. LA: light-adapted; DA: dark-adapted.

For dataset 1, healthy subjects were 4 women and 5 men. The mean age was 45 years, ranging 31 to 57 years. As there were no recordable pupil responses to blue light stimulus intensities of − 6.0 and − 5.5 log cd/m^2^ in the dark-adapted sequence of protocol 1, we analyzed the pupil responses to light intensities at − 5, − 4.5, − 4, − 3.5, − 3, − 2.5, − 2, − 1.5, − 1, 0.5, 1 and 2.65 log cd/m^2^. Collectively, these responses represent a transition from a primarily rod-driven pupil response at lower intensities to a mixed response at higher intensities due to increasing cone and melanopsin contribution. The mean pupil tracings for these 13 stimuli (stimulus–response curve) for all subjects are shown in Fig. 1A. This figure shows that the dark-adapted pupil response to a very bright blue light (2.65 log cd/m^2^) has a visibly different waveform compared to blue light stimuli having lower intensities and to the same very bright blue intensity (2.65 log cd/m^2^) we presented under light-adapted conditions. Specifically under dark-adapted conditions, following a rapid maximal constriction, the pupil does not begin to dilate quickly if the stimulus is a very bright blue light; rather the pupil tends to stay constricted after the light stimulus is removed.

**Figure 2:**
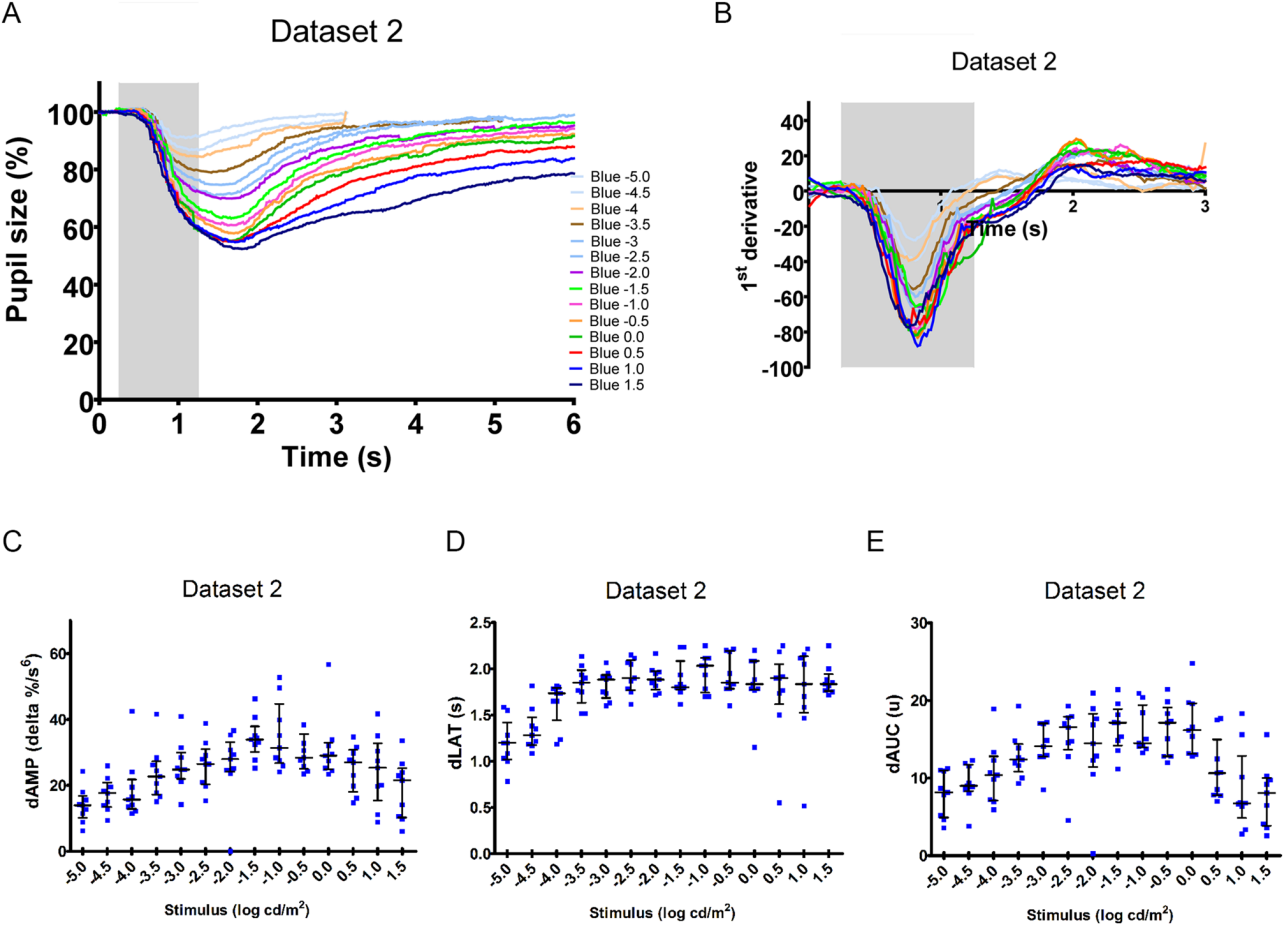
Pupil response curve and derivative analysis of Dataset 2 to blue light stimuli presented under dark-adapted condition. (**A**) The mean relative pupil size (plain line) before, during and after a 1 s light stimulus is plotted against time (response curve). The different colors represent the different stimulus intensities from − 5 log cd/m^2^ to 1.5 log cd/m^2^. (**B**) The mean derivative responses plotted for the first 3 s of the pupil recordings shown in (**A**). The shaded box represents the duration of the light stimulus. Three parameters were obtained by quantification of the derivative curve: the maximal positive peak amplitude (dAMP) (**C**), the latency of dAMP (**D**) and the area under the curve of the positive peak of the derivative (dAUC) (**E**) are presented as distribution plots. The median (long horizontal bar) and the interquartile range (short horizontal bar) are superposed at each stimulus intensity.

For dataset 2, healthy subjects were 3 males and 6 females. The mean age was 46 years, ranging 38 to 57 years. The stimulus–response curves to 14 blue light stimuli from − 5 to 1.5 log cd/m^2^ in half-log steps presented in dark-adapted conditions are shown in Fig. 2A and demonstrate similar dynamics to similar light stimuli compared to that from dataset 1 (Fig. 1A).

### Quantification of the early dynamic phase of pupillary dilation

**Figure 3:**
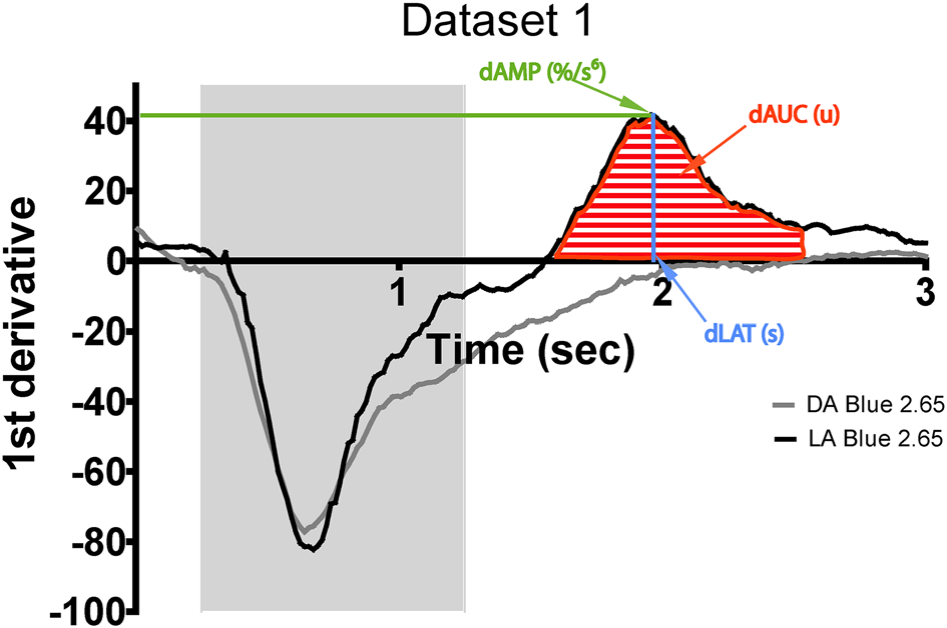
Schematic figure showing the parameters obtained from the derivative curve response. The mean derivative curve in dark-adapted (grey line) or light-adapted (black line) conditions in response to 2.65 log cd/m^2^ blue stimuli (dataset 1) is represented as a function of time. Note the difference in the shape of the curve such that grey line does not cross Y = 0. This difference illustrates how dAMP, dLAT and dAUC can discriminate between the two types of the early pupillary dilation kinetic. dAMP reflects the maximal positive peak amplitude and dLAT is its latency. The dAUC corresponds to the area under the curve of the positive peak of the derivative. These three parameters are shown for the derivative curve of the light-adapted response (black line). LA: light-adapted; DA: dark-adapted.

The derivative response curves (mean values) for dataset 1 and dataset 2 are shown in Figs. 1B and 2B respectively. At baseline, the derivative values are close to Y = 0, representing little to no pupillary movement. The maximal negative deflection is the maximal rate of change of pupillary constriction. When Y = 0 again, this corresponds to the moment when the maximal constriction is reached and there is again, briefly, no pupillary movement. On visual inspection, except the pupil response curve of the dark-adapted 2.65 log cd/m^2^, all curves intersect Y = 0 within the first 2.75 s of light onset and thereafter ascend to positive derivative values which indicate a dilation movement where the maximal positive deflection is the maximal rate of change of pupillary dilation. From the response curve to blue light 2.65 cd/m^2^ under dark-adapted condition (Fig. 1A), the pupil demonstrates a delayed and prolonged constriction. At this light stimulus, the latency to maximal constriction occurs at 1.9 ± 0.1 s. Then the pupil neither constricts further nor dilates between 2 to 3 s from stimulus onset (Fig. 1B). Consequently, its corresponding derivative curve does not cross Y = 0 within 2.75 s of light stimulus onset indicating no dilation movement in the early dynamic phase. In contrast, the derivative response curve to the same blue light 2.65 log cd/m^2^ in the light-adapted sequence (black line) does intersect the y-axis and demonstrates the greatest amplitude of the positive peak with the shortest latency, indicating the greatest change in dilation compared to all other stimuli (Fig. 3).

The dAMPs (derivative maximal positive amplitudes) for all subjects comprising datasets 1 and 2 are shown for each intensity in a scatterplot Figs. 1C and 2C and values are given in Table 1. The 3 dimmest light intensities (− 5, − 4.5 and − 4 log cd/m^2^) evoked a pupillary constriction which is very small as these intensities are near the threshold intensity (Figs. 1A and 2A), and the dilation responses are similarly weak and ill-defined (Figs. 1A and 2A) and the dAMPs are very low (Figs. 1C and 2C)^6^. To light intensities − 3.5 log cd/m^2^ and brighter, the dAMPs had a tendency to increase with stimulus intensity until − 1.0 log cd/m^2^. At the brightest blue light 2.65 log cd/m^2^ in the dark-adapted sequence, there was no observable pupillary dilation following maximal constriction (Fig. 1A). This absence of active dilation in the early dynamic phase is confirmed by an absence of positive peak amplitude on the derivative response curve (Fig. 1B) and a dAMP of zero (Fig. 1C).

**Table 1:** Quantification of derivatives of normal cohorts.

Dataset 1		dAMP (delta %/s^6^)			dLAT (s)			dAUC (u)		
Stimuli intensity (log cd/m^2^)	n	25% Perc	Median	75% Perc	25% Perc	Median	75% Perc	25% Perc	Median	75% Perc
− 5.00	9	5.7	8.9	16.4	1.4	1.6	1.7	2.1	4.7	8.5
− 4.50	9	9.3	11.6	15.3	1.4	1.7	1.8	3.9	6.7	10.2
− 4.00	9	9.0	11.8	16.6	1.7	2.1	2.3	5.2	6.1	9.2
− 3.50	9	17.5	23.5	26.0	1.8	2.0	2.1	10.0	10.6	14.1
− 3.00	9	15.3	20.7	26.9	1.8	1.9	2.1	9.0	12.2	13.6
− 2.50	9	22.0	26.7	30.3	1.7	1.9	2.1	9.8	12.2	15.8
− 2.00	9	24.2	27.4	28.1	2.0	2.1	2.1	11.8	13.4	17.4
− 1.50	9	21.7	23.4	30.2	2.0	2.0	2.2	12.6	14.9	16.7
− 1.00	9	29.1	34.1	40.1	2.0	2.0	2.2	14.3	16.3	17.4
0.50	9	29.0	32.2	38.0	2.0	2.1	2.3	15.5	16.7	18.0
1.00	9	18.0	26.1	33.5	2.1	2.2	2.4	8.3	10.9	17.5
2.65	9	− 0.1	2.3	6.5	1.9	2.2	2.4	0.0	0.4	1.1
LA 2.65	9	39.2	52.5	56.3	1.9	2.0	2.1	18.4	22.1	25.4

Dataset 2		dAMP (delta %/s^6^)			dLAT (s)			dAUC (u)		
Stimuli intensity (log cd/m^2^)	n	25% Perc	Median	75% Perc	25% Perc	Median	75% Perc	25% Perc	Median	75% Perc
− 5.0	9	10.2	13.9	16.8	1.3	1.5	1.7	4.9	8.1	11.0
− 4.5	9	13.4	17.7	20.9	1.4	1.5	1.7	8.3	9.0	11.7
− 4.0	9	12.9	15.7	21.8	1.7	2.0	2.0	7.1	10.4	12.8
− 3.5	9	17.2	22.7	27.3	1.9	2.1	2.2	10.8	12.4	14.4
− 3.0	9	22.0	24.8	30.0	1.9	2.1	2.2	12.8	14.1	17.0
− 2.5	9	20.4	26.5	31.0	2.0	2.2	2.3	13.7	16.6	17.9
− 2.0	9	24.1	28.0	33.1	2.0	2.1	2.2	11.4	14.5	18.3
− 1.5	9	30.1	33.9	37.9	2.0	2.1	2.3	14.2	17.1	18.9
− 1.0	9	26.8	31.4	44.8	2.0	2.3	2.4	13.9	14.5	19.4
− 0.5	9	25.0	28.4	35.6	2.0	2.1	2.4	12.8	17.1	19.1
0.0	9	24.9	29.1	32.9	2.0	2.1	2.3	13.1	16.2	19.7
0.5	9	18.0	27.0	30.8	1.9	2.2	2.3	7.8	10.6	15.0
1.0	9	15.5	25.4	32.8	1.8	2.1	2.4	4.8	6.8	12.9
1.5	9	10.2	21.6	25.2	2.0	2.1	2.2	3.9	8.1	10.0

Median and interquartile range (25% Percentile (Perc.) and 75% Perc.) are indicated for dAMP, dLAT and dAUC of Dataset 1 and Dataset 2.

The dLAT (derivative latency time to the maximal positive peak amplitude) is shown for all subjects of datasets 1 and 2 in scatterplots Figs. 1D and 2D respectively. The dLAT appears to be a fairly stable parameter, not intensity dependent, with medians from 1.8 to 2.0 s for all stimuli except the 2 dimmest (− 5 and − 4.5 log cd/m^2^, Table 1). We point out that the value of the dLAT is measured from the onset of light stimulus whereas the derivative response curves of Figs. 1B and 2B begin with 250 ms of baseline before the stimulus onset. As the dLATs for the 2 dimmest stimuli were generally shorter and/or highly variable compared to the other stimuli, we considered the responses to these 2 dimmest stimuli not reliable and below the threshold for quantification for all further analysis.

The dAUC (derivatives area under the curve of the positive peak) is shown in a scatterplot as a function of stimulus intensity (Figs. 1E and 2E). Because we had identified the dLATs to the 2 dimmest stimuli (− 5 and − 4.5 log cd/m^2^) as non-valid, we used the mean AUC of these 2 dimmest stimuli to determine a minimum AUC value which was considered valid for this study. The minimum AUC is 10 (in arbitrary units). An AUC equal to or greater than 10 indicates a rapid pupillary dilation movement following a light-evoked pupillary constriction (Figs. 1E and 2E).

For dataset 1, dAUCs of dark-adapted responses increased with increasing stimulus intensity until a maximum dAUC at 0.5 log cd/m^2^. From 1 log cd/m^2^ dAUC values decreased and became negligible at 2.65 log cd/m^2^ (< 10 arbitrary units), consistent with the observed sustained pupillary constriction observed at this bright intensity (Fig. 1A). Moreover, the maximal dAUC value is presented by the response to the light-adapted blue light 2.65 log cd/m^2^, consistent with the tracing shown in Fig. 1B. For dataset 2, dAUC values increased with light intensity and did not differ significantly at intensities between − 2.5 to 0.0 log cd/m^2^ (Fig. 2E). Thereafter, dAUC values from 0.5 to 1.5 log cd/m^2^ decreased gradually and became negligible at 1.5 log cd/m^2^. Together the dAUC of dataset 1 and 2 revealed the change in the dilation dynamic of the pupil with increasing blue light intensity in dark-adapted conditions.

If we consider collectively the early phase dynamic quantification of dataset 1 and 2 from blue lights presented in dark-adapted condition, we find that dAMP and dAUC increase with increasing light intensity until − 1.0 log cd/m^2^ and then progressively decrease at higher intensities to 1.5 log cd/m^2^. At 2.65 log cd/m^2^, the dAMP and dAUC values are negligible. In contrast, dLAT reaches a median value of 1.8 s at intensity around − 4.0 log cd/m^2^ and remains constant despite increasing light intensity to 2.65 log cd/m^2^.

## Discussion

This study applied a derivative analysis of the early pupillary dilation dynamics developed from pupillographic tracings in murine models to pupil recordings of human eyes. The aim of this work is to determine if such an analysis can provide new objective metrics for potentially grading the outer photoreceptor contribution to the pupil light response in humans. We previously observed in a mouse model for outer retina degeneration that the dynamics of the early phase of pupillary dilation (first 3 s) after pupillary constriction to a light stimulus changed gradually with increasing age of the mice, indicating that such change follows the progression of rod and cone loss (Kostic C et al., IOVS 2015; 56: ARVO E-Abstract 574). In another study using murine models, we noticed that the early phase dynamic in mice deprived of rods and cones is notably different compared to wild-type mice^12^. Specifically, in rod-less and cone-less mice, pupil constriction was observed to all blue stimuli between 0.6 and 2 log cd/m^2^ but only to bright red stimuli of 4.5 log cd/m^2^ but the pupillary constriction was delayed and prolonged without any dilation in the early phase. This demonstrated the impact of the outer retinal function on the early dynamic of the pupillary dilation for rod or cone-weighted stimuli. Moreover other studies using pharmacologic agents blocking rod and cone activity in rat and macaque models have shown that the influence of rods and cones following offset of selected stimuli overrides the slow melanopsin pathways^1,14^.

In this study of healthy eyes, we analyzed a series of dark-adapted pupil responses to blue light intensities ranging from near threshold to suprathreshold using two slightly different stimulus protocols (dataset 1 and dataset 2). For both datasets we found that dAMP and dAUC increase when the light stimulus increases from − 5.0 log cd/m^2^ until − 1.0 log cd/m^2^. The presence of dAMP, which is a measure of rapid dilation, to low blue stimuli in dark conditions suggests a predominant rod activation consistent with previous studies which also shows rod contribution to the pupil responses for light intensities below − 1.0 log cd/m^2^^3,15^. Adhikari et al. analyzed the post-illumination light response and reported rod input to this pupil response up to 1.7 s after the light offset^16^. This time period is similar to the time window used in our study for the derivative analysis (0.25 to 2.25 s after the light onset which corresponds to < 1.25 s after the light offset). Increasing light intensity increases recruitment of rods but then also of cones, amplifying their influence on both constriction and dilation. From − 0.5 log cd/m^2^, dAMP and dAUC progressively decrease at higher intensities reaching negligible values at 2.65 log cd/m^2^. Presumably, this is related to increasing activation of melanopsin, masking rod and cone contribution, and reflecting its slower kinetic^14,17^ on the pupil response. In contrast to dAMP and dAUC, dLAT from − 4.0 log cd/m^2^ remains constant (median 1.8 s) with increasing intensity.

The dark-adapted pupil response curve shows a significant change in the early dilation dynamics when the light stimulus reaches 2.65 log cd/m^2^ which is well illustrated by the absence of dAMP and dAUC. This kinetic is consistent with that observed in rodless and coneless mice^12^ or in retina blocked for outer retina function^1,14^, both conditions in which melanopsin is the dominant influence on the pupil light response. Interestingly, the same bright blue light of 2.65 log cd/m^2^, when presented under light adaptation, does not show the same pupil response as under dark adaption. In fact, the light-adapted dAMP and dAUC values were the highest recorded in this study, suggesting that cones are the main contributor to the control of the dilation under light-adapted conditions. To further support our hypothesis we performed a similar post-hoc analysis of responses to red stimuli under light-adapted conditions and this also showed high dAMP and dAUC values (Supplementary Fig. S1). Similar kinetics of the early dilation have been observed in other studies when applying cone weighted stimuli^3,18,19^. Indeed, in controls, we found that stimuli of 1 or 1.5 log cd/m^2^ drive an intermediate type of dilation with dAUC that is significantly lower than the response to − 3.0 to 0 log cd/m^2^ but not null. This result is in accordance with the data published by Park et al. which shows a progressive delay in the dilation when increasing blue stimuli from − 4 to 2.6 log cd/m^2^^3^. The dAMP and dAUC metrics we developed in this study appear to adequately quantify these two different dilation kinetics.

In order to better understand the potential utility of this pupil analysis in the clinical setting, we examined the pupillometry results of 3 patients with outer retina dysfunction who had been tested with a similar protocol as our healthy subjects. We observed that massive loss of rod function, as determined from electroretinography, was associated with a more rapid pupillary dilation in the early phase and this was best observed at stimuli 1.0 and 1.5 log cd/m^2^ (Supplementary Fig. S2). In addition, the maximal dAUC and dAMP values for all 3 patients were at the 95th percentile limit of the values from healthy subjects. Unexpectedly, these results from the 3 patients contrast to what was observed in rod-deficient mice which showed a less rapid dilation rate. One hypothesis for the unexpectedly fast dilation rate in these patients is that, under normal condition, rods participate to the inhibition of the early dilation in response to these stimuli and that this inhibitory effect is lost in outer retinal degeneration. This mechanism might simply be absent in the mouse retina. Also we recognize that there are differences in the anatomy of the human retina and mouse retina, such as cone type and distribution, and anatomic differences could implicate different type of retinal circuitry for pupil control^20^. Moreover, the dilator muscle in mice is an underdeveloped structure compared to that of humans which is more robust and mechanical differences in the muscular force could limit the importance of its action during the dilation phase in mice^21–23^. In addition we cannot exclude regional preservation of rods in these patients^24^ which could still contribute to the pupil response and explain why dAUC and dAMP are at the limit of the healthy subject values. Further studies with a larger number of patients with well characterized retinal outer dysfunction will be essential to evaluate the utility of the derivative analysis in clinical chromatic pupillometry.

In summary, our study describes an analysis of the early pupillary dilation kinetics to identify quantifiable parameters which can be related to outer versus inner photoreceptor activity. We found that dAMP and dAUC measured at near-threshold light intensities up to − 1.0 log cd/m^2^ reflect increasing activation of rods and cones. A maximal value is obtained at − 1.0 log cd/m^2^ when the rate of change of dilation is maximal and this light intensity might be an important stimulus for clinical pupil protocols intending to activate the outer photoreceptors. Furthermore, the decreasing dAMP and dAUC from light intensities above − 0.5 log cd/m^2^ suggest that melanopsin may be a potential additional contributor to the pupil response, even at relatively moderate intensities. However at 2.65 log cd/m^2^ blue light, the melanopsin appears to be the main driver of the pupil response masking the outer photoreceptor input and we cannot distinguish, using the early pupillary dilation analysis, any activity of rods and cones at this bright intensity. The analysis of 3 patients with outer retinal disease suggests that a differential response of the early pupillary dilation kinetics may be a way to detect loss of rod and cone activity. Further studies are anticipated to establish reliable normative values of the dAMP and dAUC which may be used for clinical evaluation of patients with retinal dysfunction.

## Methods

Informed consent was obtained and signed by all healthy subjects and patients after explanation of the nature and possible consequences of the study. All methods were carried out in accordance with approved protocols of Montpellier University Hospital, and in agreement with the Declaration of Helsinki. The Ministry of Public Health accorded approval for biomedical research under the authorization number 11018S. The raw data for this study is comprised of original pupil recordings (n = 21) collected at a single institute, the Gui de Chauliac Hospital, Institut des Neurosciences de Montpellier in Montpellier, in 2011 over a 6 month period using two different light stimulus protocols. The recordings from the healthy group tested with the first protocol (n = 9) were analyzed for the maximum pupillary contraction amplitude and the results were published^6^. The additional recordings of healthy controls and patients with outer retinopathy with the second (pilot) protocol have never been published. We were interested to perform a derivative analysis of the pupillary dynamics of these recordings because both light stimulus protocols used multiple (14) light intensities, permitting an analysis over an 8 log-unit range of intensities. The derivative analysis in this study assessed the early rapid pupillary dilation that immediately follows maximum contraction and such an analysis has not been previously applied to human pupillographic recordings.

The pupillometer was a ColorDome Ganzfeld ERG apparatus (Diagnosys, Lowell, Mass) linked to a dual channel binocular eye frame pupil recorder (Arrington Research, Scottsdale, AZ) which recorded pupil diameter continuously at 30 Hz. The testing distance from the front of the eye to the bowl was fixed at 75 mm with a horizontal radius of the viewing angle of 45°. The untested eye was occluded with a patch and the tested (stimulated) eye was also the monitored eye.

The raw pupil tracings of 11 healthy subjects tested in April 2011 with protocol 1 (see below) served as the control group in a previously published study^6^. For this present study, a derivative analysis could not be reliably performed on two of these recordings; thus remaining 9 recordings comprised our dataset 1 of the current study. The light stimulus protocol (protocol 1) for dataset 1 included a dark- and light-adapted sequence of red lights (640 ± 10 nm) and blue lights (467 ± 17 nm). Each light stimulus was 1-s in duration. The light stimulus was presented to the right pupil which was recorded continuously. In the dark-adapted sequence (dark adaptation 10 min, 0 cd/m^2^), the pupil was recorded continuously for 10 s of total darkness and then in response to a series of alternating blue and red light stimuli, starting at − 6.0 log cd/m^2^ and increasing in half-log steps to − 1.0 log cd/m^2^, and then at intensities of 0.5, 1.0 and 2.65 log cd/m^2^. For the light-adapted sequence, the subject was first light adapted to room light (10 min) and then positioned in the Ganzfeld bowl for adaptation to blue light (3 min, 0.78 log cd/m^2^).This blue light in the bowl served as the background light upon which the red-light stimuli were presented in increasing intensity from − 1.0 to 1.5 log cd/m^2^ with half log-unit steps followed by a red light stimulus at 2.65 log cd/m^2^. At the end of the red light stimuli, the blue background was maintained and blue light stimulus (1 s) at 2.65 log cd/m^2^ was superimposed.

The pupil recordings from a second group of 9 healthy adults aged 38 to 57 with normal eyes had been tested with another light stimulus protocol (protocol 2) of 14 light stimulus intensities ranging from − 6.0 log cd/m^2^ to 1.5 log cd/m^2^ (0 log = 1 cd/m^2^) (unpublished data). These 9 recordings comprised our dataset 2 for the current study. The dark-adapted sequence of protocol 2 presented first blue stimulus lights from − 6.0 log cd/m^2^ to 1.5 log cd/m^2^ in increasing half-log steps and then red stimulus lights from − 3.5 log cd/m^2^ to 1.5 log cd/m^2^ in increasing half-log steps.

In this study, we selected for analysis the pupil responses to blue light stimuli in dark-adapted conditions in order to have a basis for comparison to a similar analysis of blue light stimuli in healthy and retinal degenerate knockout mice^12^. We also included in this analysis the light-adapted response to the brightest blue stimulus 2.65 log cd/m^2^ from the light-adapted sequence. For all recordings, pupil size was converted to relative pupil size as a function of baseline pupil size. The baseline pupil size was determined as the mean pupil size in the 250 ms before the first light stimulus. The relative pupil size was plotted as a function of time for 6 first seconds of each light stimulus i.e., from 250 ms before light stimulus onset to 5.75 s following light stimulus onset.

For each stimulus, a derivative analysis was performed (first derivative, 6 orders, 12th neighbors, GraphPad Prism 5.01 software) of the relative pupil size during the early dynamic phase. We defined the early dynamic phase as the first 2.75 s from light onset, as based on our previous experience with derivative analysis in normal and knockout mice^12^. We then plotted the derivative as a function of time from 250 ms before light stimulus onset to 2.75 s following light onset to obtain a derivative response curve. The derivative response curve describes the change in the pupillary movement, a negative value indicating decreasing pupil size (constriction) and a positive value indicating increasing pupil size (dilation). We distinguish two peak values on the derivative curve: a minimal value (negative peak) and a maximal value (positive peak). These two peak values represent a maximum variation of constriction (negative peak value) or of dilation (positive peak value). As pupillary movement slows down, for example when the constriction stabilizes briefly at minimum pupil size, the derivative value returns to zero. If the pupil dilates from minimum pupil size, the derivative value becomes positive.

We measured the following parameters from the derivative response curve: the maximal positive peak amplitude (dAMP), the latency to the maximal positive peak amplitude (dLAT) and the area under the curve of the positive peak (dAUC, Fig. 3). We defined dAMP as the maximal positive value between 0.5 and 2.25 s after light onset and dLAT as the time from light onset to the time of dAMP. We set the limits for the area under the curve for the positive derivative between 0.25 and 2.25 s after light onset based on the more pronounced derivative curve obtained in response to the highest stimuli 2.65 log cd/m^2^ in light-adapted conditions which is used as control for cone main input (see discussion). This time period ensured inclusion of the positive peak from Y = 0 and limited the quantification of dAUC to the first positive peak of the derivative response curve. dAUC was calculated using excel software as the sum of (0.0167 s X mean y value between two successive time points when y > 0) from 0.25 s to 2.25 s after light onset. One-way ANOVA were performed and Bonferroni post-hoc test compared, within dataset 1 or dataset 2, dAMP, dLAT and dAUC values for different stimulus intensity (Supplementary Table S1 and S2).

## Supplementary Information

Supplementary Information.
