# Supplementary material for: Quantification of the early pupillary dilation kinetic to assess rod and cone activity
Source: Sci Rep. 2021 May 5;11:9549. doi: 10.1038/s41598-021-88915-z (PMC8099900; doi:10.1038/s41598-021-88915-z)

Quantification of the early pupillary dilation kinetic to assess rod and cone activity.

Kostic Corinne[1], Crippa Sylvain [1,2], Leon Lorette[2], Hamel Christian[3], Meunier Isabelle[3] and Kawasaki Aki[2]*

[1] Group for Retinal Disorder Research, Department of Ophthalmology, University Lausanne, Hôpital Ophtalmique Jules Gonin, Switzerland

[2]Neuro-ophtalmology, Department of Ophthalmology, University Lausanne, Hôpital Ophtalmique Jules Gonin, Switzerland

[3] Institute for Neurosciences of Montpellier INSERM U1051, University of Montpellier, Montpellier, France; Centre de Référence pour les Maladies Sensorielles Génétiques, Hôpital Gui de Chauliac, CHRU Montpellier, 34090 Montpellier, France.

Supplementary information

Supplementary Table S1. Statistical analysis of comparison of the derivatives values of dataset 1 for different light stimuli. One-way ANOVA analysis followed by Bonferroni post-hoc test were performed to compare, within dataset 1, dAMP, dLAT and dAUC values for different stimulus intensity. T-values are indicated in italic when $p > 0.05$: in bold p values are significant: in red $p<0.001$, blue $p<0.01$, black $p<0.05$

dAMP

Stimuli	-5.00	-4.50	-4.00	-3.50	-3.00	-2.50	-2.00	-1.50	-1.00	0.50	1.00	2.65
-5												
-4.5	*0.04*											
-4	*0.12*	*0.09*										
-3.5	*3.07*	*3.04*	*2.95*									
-3	*2.79*	*2.75*	*2.66*	*0.28*								
-2.5	**4.25**	**4.21**	**4.12**	*1.18*	*1.46*							
-2	**4.37**	**4.34**	**4.25**	*1.30*	*1.58*	*0.12*						
-1.5	**3.92**	**3.88**	**3.79**	*0.85*	*1.13*	*0.33*	*0.45*					
-1	**6.52**	**6.49**	**6.40**	*3.45*	**3.73**	*2.27*	*2.15*	*2.60*				
0.5	**7.13**	**7.10**	**7.01**	**4.06**	**4.34**	*2.89*	*2.76*	*3.22*	*0.61*			
1	**4.38**	**4.34**	**4.25**	*1.31*	*1.59*	*0.13*	*0.01*	*0.46*	*2.14*	*2.76*		
2.65	*2.72*	*2.75*	*2.84*	**5.79**	**5.51**	**6.97**	**7.09**	**6.64**	**9.24**	**9.85**	**7.10**	
LA 2.65	**11.11**	**11.07**	**10.98**	**8.04**	**8.32**	**6.86**	**6.74**	**7.19**	**4.59**	**3.98**	**6.73**	**13.83**

dLAT

Stimuli	-5.00	-4.50	-4.00	-3.50	-3.00	-2.50	-2.00	-1.50	-1.00	0.50	1.00	2.65
-5												
-4.5	*1.87*											
-4	**4.61**	*2.74*										
-3.5	**4.16**	*2.29*	*0.45*									
-3	*3.32*	*1.45*	*1.29*	*0.84*								
-2.5	**3.74**	*1.87*	*0.87*	*0.42*	*0.42*							
-2	**5.30**	*3.43*	*0.68*	*1.14*	*1.97*	*1.55*						
-1.5	**5.23**	*3.36*	*0.62*	*1.07*	*1.90*	*1.49*	*0.07*					
-1	**5.63**	**3.76**	*1.02*	*1.47*	*2.31*	*1.89*	*0.33*	*0.40*				
0.5	**6.15**	**4.28**	*1.54*	*1.99*	*2.82*	*2.41*	*0.85*	*0.92*	*0.52*			
1	**6.35**	**4.48**	*1.74*	*2.19*	*3.02*	*2.61*	*1.05*	*1.12*	*0.72*	*0.20*		
2.65	**5.88**	**4.01**	*1.27*	*1.72*	*2.56*	*2.14*	*0.58*	*0.65*	*0.25*	*0.27*	*0.47*	
LA 2.65	**4.56**	*2.69*	*0.05*	*0.40*	*1.24*	*0.82*	*0.74*	*0.67*	*1.07*	*1.59*	*1.79*	*1.32*

dAUC

Stimuli	-5.00	-4.50	-4.00	-3.50	-3.00	-2.50	-2.00	-1.50	-1.00	0.50	1.00	2.65
-5												
-4.5	*1.41*											
-4	*1.67*	*0.26*										
-3.5	**4.67**	*3.25*	*3.00*									
-3	**4.98**	**3.57**	*3.31*	*0.32*								
-2.5	**5.56**	**4.14**	**3.88**	*0.89*	*0.57*							
-2	**6.77**	5.36	5.10	*2.11*	*1.79*	*1.22*						
-1.5	**6.86**	5.45	5.19	*2.19*	*1.88*	*1.31*	*0.09*					
-1	**8.19**	6.78	6.52	*3.53*	*3.21*	*2.64*	*1.42*	*1.33*				
0.5	**9.36**	7.95	7.69	4.69	**4.38**	**3.81**	*2.59*	*2.50*	*1.17*			
1	**5.28**	**3.86**	**3.61**	*0.61*	*0.29*	*0.28*	*1.50*	*1.58*	*2.92*	**4.08**		
2.65	*3.07*	**4.49**	**4.75**	**7.74**	**8.06**	**8.63**	**9.85**	**9.93**	**11.27**	**12.43**	**8.35**	
LA 2.65	**12.12**	**10.70**	**10.44**	**7.45**	**7.13**	**6.56**	**5.34**	**5.26**	**3.92**	*2.76*	**6.84**	**15.19**

Supplementary Table S2. Statistical analysis of comparison of the derivatives values of dataset 2 for different light stimuli. One-way ANOVA analysis followed by Bonferroni post-hoc test were performed to compare, within dataset 2, dAMP, dLAT and dAUC values for different stimulus intensity. T-values are indicated in italic when $p > 0.05$: in bold p values are significant: in red $p<0.001$, blue $p<0.01$, black $p<0.05$

dAMP

Stimuli	-5.0	-4.5	-4.0	-3.5	-3.0	-2.5	-2.0	-1.5	-1.0	-0.5	0.0	0.5	1.0
-5													
-4.5	0.85												
-4	1.27	0.43											
-3.5	2.53	1.69	1.26										
-3	3.12	2.27	1.85	0.59									
-2.5	3.23	2.38	1.96	0.70	0.11								
-2	3.11	2.26	1.84	0.58	0.01	0.12							
-1.5	**5.28**	**4.43**	**4.01**	2.75	2.16	2.05	2.17						
-1	**5.46**	**4.61**	**4.18**	2.92	2.34	2.23	2.35	0.18					
-0.5	**5.11**	**4.26**	**3.84**	2.58	1.99	1.88	2.00	0.17	0.35				
0	**4.50**	**3.65**	3.23	1.97	1.38	1.27	1.39	0.78	0.96	0.61			
0.5	2.86	2.01	1.58	0.33	0.26	0.37	0.25	2.42	2.60	2.25	1.64		
1	2.72	1.87	1.44	0.19	0.40	0.51	0.39	2.56	2.74	2.39	1.78	0.14	
1.5	1.26	0.41	0.01	1.27	1.86	1.97	1.85	**4.02**	**4.20**	**3.85**	3.24	1.60	1.46

dLAT

Stimuli	-5.0	-4.5	-4.0	-3.5	-3.0	-2.5	-2.0	-1.5	-1.0	-0.5	0.0	0.5	1.0
-5													
-4.5	1.00												
-4	2.89	1.90											
-3.5	**4.25**	3.26	1.36										
-3	**4.28**	3.28	1.38	0.02									
-2.5	**4.93**	**3.93**	2.03	0.67	0.65								
-2	2.94	1.95	0.05	1.31	1.33	1.98							
-1.5	**4.70**	**3.70**	1.81	0.45	0.42	0.22	1.76						
-1	**5.20**	**4.20**	2.31	0.95	0.92	0.27	2.26	0.50					
-0.5	**5.00**	**4.00**	2.11	0.75	0.72	0.07	2.06	0.30	0.20				
0	**4.38**	3.38	1.48	0.12	0.10	0.55	1.43	0.32	0.82	0.62			
0.5	**3.73**	2.73	0.84	0.52	0.55	1.20	0.79	0.97	1.47	1.27	0.65		
1	**3.68**	2.68	0.79	0.57	0.60	1.25	0.74	1.02	1.52	1.32	0.70	0.05	
1.5	**4.60**	**3.60**	1.71	0.35	0.32	0.32	1.66	0.10	**0.60**	**0.40**	0.22	0.87	0.92

dAUC

Stimuli	-5.0	-4.5	-4.0	-3.5	-3.0	-2.5	-2.0	-1.5	-1.0	-0.5	0.0	0.5	1.0
-5													
-4.5	0.90												
-4	1.79	0.90											
-3.5	3.30	2.40	1.51										
-3	4.13	3.23	2.33	0.83									
-2.5	**4.72**	**3.82**	2.93	1.42	0.59								
-2	**3.95**	3.06	2.16	0.65	0.17	0.77							
-1.5	**5.69**	**4.79**	**3.90**	2.39	1.57	0.97	1.74						
-1	**5.42**	**4.52**	**3.62**	2.12	1.29	0.69	1.46	0.28					
-0.5	**5.57**	**4.68**	**3.78**	2.27	1.45	0.85	1.62	0.12	0.16				
0	**5.91**	**5.01**	**4.11**	2.61	1.78	1.18	1.95	0.21	0.49	0.33			
0.5	2.13	1.23	0.33	1.17	2.00	2.59	1.82	3.56	3.29	3.44	**3.78**		
1	0.41	0.49	1.38	2.89	**3.72**	**4.31**	3.54	**5.28**	**5.00**	**5.16**	**5.49**	1.72	
1.5	0.11	1.01	1.91	3.41	**4.24**	**4.83**	**4.07**	**5.80**	**5.53**	**5.68**	**6.02**	2.24	0.52

Supplementary Figure S1. Pupil response curve and derivative analysis of Dataset 1 to red light stimulis presented under light-adapted condition. (A) The mean relative pupil size (plain line) before, during and after a 1 second light stimulus is plotted against time (response curve). The different red levels represent the different stimulus intensities from -1.0 log cd/m^2 to 2.65 log cd/m^2. (B) The mean derivative responses plotted for the first 3 s of the pupil recordings shown in (A). The shaded box represents the duration of the light stimulus. Three parameters were obtained by quantification of the derivative curve: the maximal positive peak amplitude (dAMP) (C), the latency of dAMP (D) and the area under the curve of the positive peak of the derivative (dAUC) (E) are presented as distribution plots. The median (long horizontal bar) and the interquartile range (short horizontal bar) are superposed at each stimulus intensity.

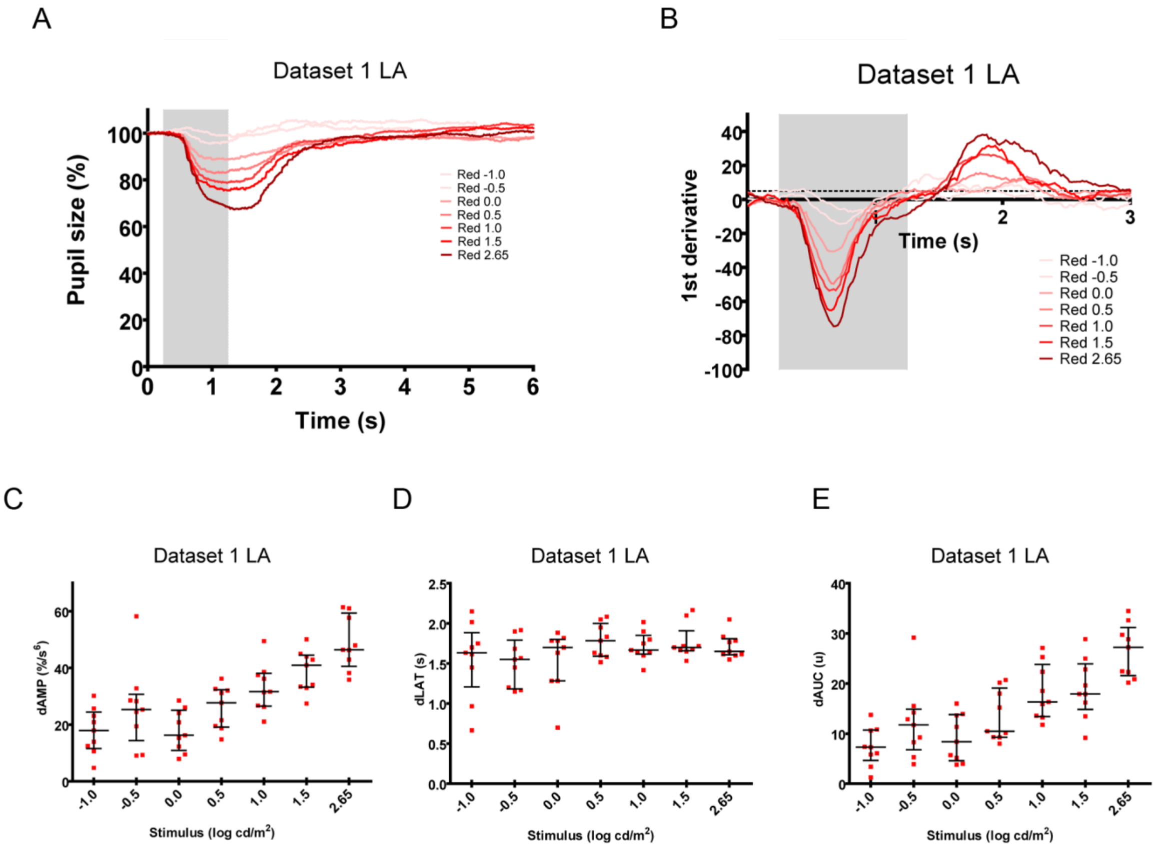

Supplementary Figure S2. Pupil response curve (a and b) and derivative response curve (c and d) to blue light (1.0 and 1.5 log cd/m^2) presented under dark-adaptation in 3 patients (P1-3) and controls (dataset 2). P1 and P2 presented a phenotype of unilateral retinitis pigmentosa (RP) and P3 was diagnosed for a congenital stationary night blindness (CSNB). The mean of control eyes are shown as solid blue line. From the response curves (a and b), the healthy eye of P1 and P2 (black and grey dotted lines) have similar dilation than controls. The affected eye of P1 (black solid line) and P2 (grey solid line) and P3 (red plain line) show a smaller constriction amplitude and faster dilation on the stimulus response curve. This is easily verified on the derivative response curve as dAMP is notably larger than control eyes and healthy eyes of P1 and P2 who have unilateral RP.

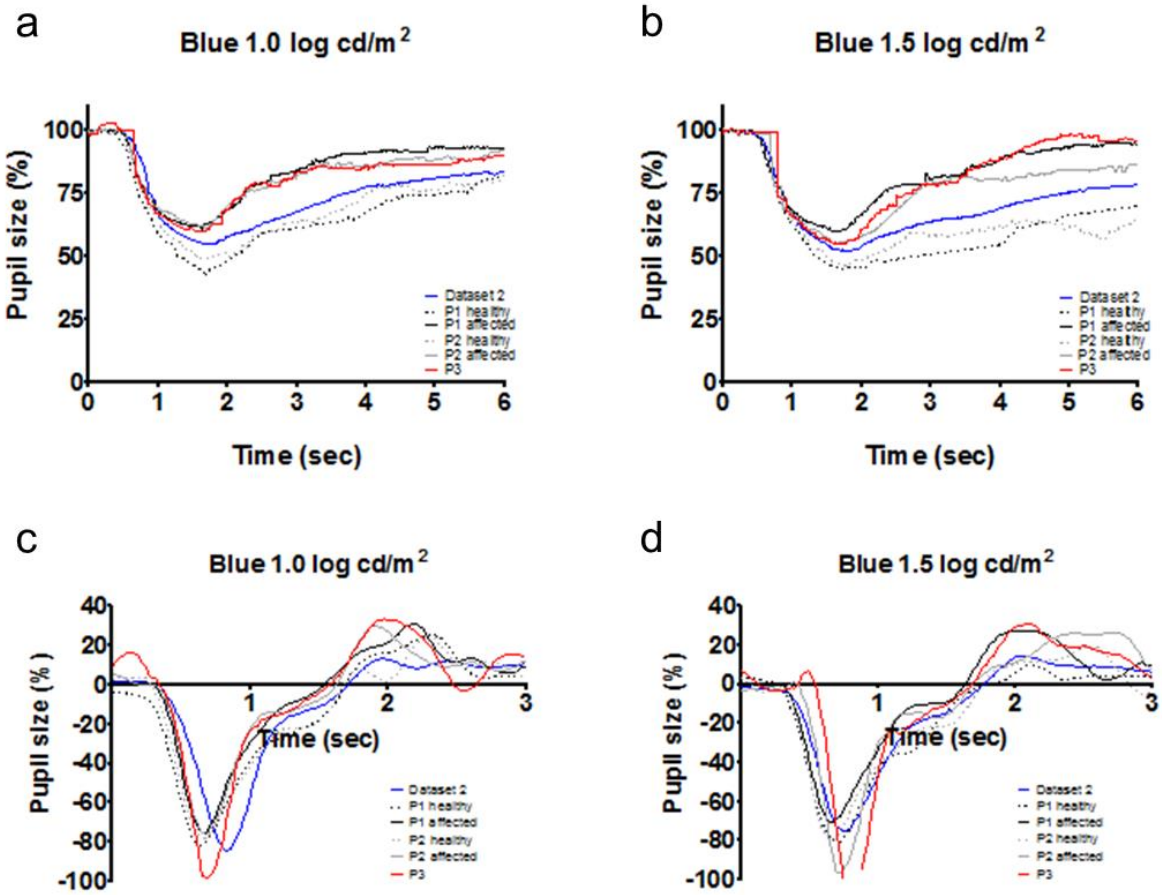

Supplement: Supplementary file 1 — Supplementary Information. [file 41598_2021_88915_MOESM1_ESM.pdf]
